# SINE Retrotransposon variation drives Ecotypic disparity in natural populations of *Coilia nasus*

**DOI:** 10.1186/s13100-019-0198-8

**Published:** 2020-01-08

**Authors:** Dong Liu, Jinquan Yang, Wenqiao Tang, Xing Zhang, Clay Matthew Royster, Ming Zhang

**Affiliations:** 1 Key Laboratory of Marine Animal Taxonomy and Evolution, Shanghai Universities, Shanghai, 201306 China; 2 Department of Epidemiology and Biostatistics, University of Georgia, Athens, GA 30602 USA; 3 Key Laboratory of Exploration and Utilization of Aquatic Genetic Resources, Ministry of Education, Shanghai, 201306 China; 4 National Demonstration Center for Experimental Fisheries Science Education, Shanghai Ocean University, Shanghai, 201306 China

**Keywords:** Ecotypic diversity, Retrotransposable element, Short interspersed nuclear elements, Long interspersed nuclear elements, Natural selection

## Abstract

**Background:**

SINEs are a type of nonautonomous retrotransposon that can transpose from one site to be integrated elsewhere in an organism genome. SINE insertion can give rise to genetic variants and regulate gene expression, allowing organisms to acquire new adaptive capacity. Studies on this subject have focused on the impacts of SINEs on genes. However, ecological disparities in fish have not yet been explained by SINEs.

**Results:**

New SINEs were isolated from *Coilia nasus,* which has two ecotypes—migratory and resident—that differ in their spawning and migration behaviors. The SINEs possess two structures that resemble a tRNA gene and a LINE retrotransposon tail. Comparison of olfactory tissue transcriptomes, intact SINE transcript copies were detected in only the migratory fish at the initial retrotransposition stage. The SINE DNA copy numbers were higher in the resident type than in the migratory type, while the frequency of SINE insertion was higher in the migratory type than in the resident type. Furthermore, SINE insertions can lead to new repeats of short DNA fragments in the genome, along with target site duplications. SINEs in the resident type have undergone excision via a mechanism in which predicted cleavage sites are formed by mutations, resulting in gaps that are then filled by microsatellites via microhomology-induced replication.

**Conclusions:**

Notably, SINEs in the resident type have undergone strong natural selection, causing genomic heteroplasmy and driving ecological diversity of *C. nasus*. Our results reveal possible evolutionary mechanisms underlying the ecological diversity at the interface between SINE mobilization and organism defense.

## Introduction

Short interspersed elements (SINEs) are a type of retrotransposon frequently found in eukaryotic genomes; these elements can expand in the genome and generate multiple copies [1, 2]. Some inserted copies may directly affect the functions of individual genes via regulation of expression or creation of novel genes in response to environmental challenges [3]. SINE insertions have been shown to benefit *Drosophila melanogaster* during the spread of this organism out of Africa [4]. SINE insertions upstream of genes can enhance gene expression and expand gene function [5]. Two functional enhancers in the *POMC* gene originated from ancient insertions in *D. melanogaster* [6]. In addition, a transposon-originated gene associated with high-latitude adaptation was identified in soybean plants [7]. Moreover, the industrial melanism of the peppered moth in Britain resulted from transposon insertion within a gene intron and provides a visible demonstration of an evolutionary response to environmental change [8].

SINEs have contributed to species evolution. Mobilization and nonhomologous recombination of SINEs have generated intraspecific polymorphisms and led to interspecific diversity [9]. The well-studied formation events of salmonid species were found to be correlated with a burst in the dispersion of retrotransposons [10]. In salmon, the mobilization of these SINEs remains ongoing and continues to drive the genomic diversity of the species [11]. In two closely related puffer fish species, transposable elements are responsible for genome size variation, with 2% SINE content in one species and 0.2% in the other [12]. The abundance and diversification of transposable elements are among the major mechanisms driving genomic variation in teleosts [13].

SINEs originated from tRNAs, 7SL RNA or 5S rRNA [2]. A majority of reported SINEs were derived from tRNAs and consist of three regions: a tRNA-related region, a body and a tail region [2]. SINEs are a type of nonautonomous retrotransposon, and in RNA form, SINEs acquire their mobility and the ability to undergo bursts of retrotransposition from long interspersed element (LINE)-encoded proteins [14]. This protein recognizes the 3′ tail sequence of the SINE, which is similar to that of a LINE [15], in a procedure that is required for both initiation of reverse transcription of the SINE RNA and integration of the SINE into a new genomic location. SINEs can integrate into gene-dense regions, into specific regions or throughout the genome [1].

SINE insertion may be deleterious to the organism genome [9]. However, these harmful insertions can be eliminated by multiple mechanisms in organism, such as breeding systems [16], purifying selection [17], ectopic recombination [18], and genetic drift [19]. The retrotransposons themselves, via mutation, can evolve to form a “fossil” state that is no longer mobile. In particular, the repeats within the 3′ tail regions of SINEs shrink rapidly [20]. Furthermore, the promoter in the tRNA-related region can accumulate mutations to disrupt the initiation of SINE transcription. The changes in SINE length, caused by random deletions can alter SINE RNA folding, leading to loss of mobility [2].

Considering the evolutionary importance of SINEs in the generation of genomic diversity, it is reasonable to consider SINEs as suitable genetic markers in systematic biology and as a tool to track common ancestry among specific taxa [21, 22]. It is widely accepted that the evolution of SINEs is unidirectional and irreversible [23], and SINE markers are homoplasy free [21]. Therefore, information regarding the ancestral states (present or absent) of the SINE-inserted loci can be very useful for phylogenetics at both the species and population levels. The application of characterization of SINE insertions as markers has been largely limited to humans, primates, whales, and a few fish species [24–26]. The major obstacles impeding broader application of SINE insertions include both isolation of SINEs from organisms for which genomic sequences are unknown and identification of a large number of polymorphic loci in genomes [21].

Currently, the retrotransposons associated with life history disparity in natural animal populations remain poorly studied*.* Our study subject, *Coilia nasus*, is an anadromous fish that has undergone rapid ecotype divergence following population expansion from the ocean to fresh water [27]. Two ecotypes of *C. nasus—*the resident type and the migratory type—are found in the Yangtze River in China, and these ecotypes differ in their spawning/migration behavior and exhibit distinct morphological divergence. During the spawning season from March to August, *C. nasus* migrate from coastal water up to the Yangtze River, even penetrate 1400 km upstream for breeding. The gonads of the fish are developing via migration, and matured fish spawn in the reaches of these rivers and adjacent lakes. After reproduction, these fish and their progeny migrate back to the ocean from September to November of the same year. Their phenotypic differences include body shape, vertebral counts, anal fin counts, eye size and gill raker counts. In particular, the maxilla of resident type is short and does not reach the edge of the gill cover, while the migratory type has a long maxilla that extends well beyond the gill cover [28]. A high level of genetic diversity has also been observed between the two types via mitochondrial DNA marker analyses [29, 30].

*C. nasus* is an economically important fish: catches of the migratory type were greater than 3000 tons in the lower reaches of the Yangtze River and accounted for 35–50% of the total fish catches in this area in the 1970s. However, the catches sharply decreased to 50 tons in 2005 [31]. The migratory fishes have been protected and the Chinese government stopped issuing special fishing licenses for this ecotype in 2019. In contrast, catches of the resident type from lakes increased from 640 tons in 1952 to more than 20,000 tons in 2004, making *C. nasus* the dominant species (~ 64% of the total fish catches) [32].

The Yangtze River is ~ 6000 km long, with some reaches and lakes interwoven. The overlapping habitats between the migratory and resident types of *C. nasus* in the Yangtze River have created challenges for the determination of gene flow between these two types. Molecular techniques have offered the opportunity to identify and delineate fish population structures that may not be apparent based on phenotypic or behavioral characteristics alone. The two ecotypes of *C. nasus* in the Yangtze River provide a good system for the study of SINE biology and determination of whether and how mobile elements have influenced the population ecology.

In this study, we selected representative samples from the collected 1200 samples, including both *C. nasus* ecotypes, which exhibit different reproductive behaviors. We examined the insertion patterns of 71 SINE loci to better understand the life history diversity of *C. nasus*. Our results provide strong evidence that SINE motility has driven the genomic heterogeneity of *C. nasus* and is associated with ecological diversity.

## Materials and methods

### Sample collection

**Fig. 1:**
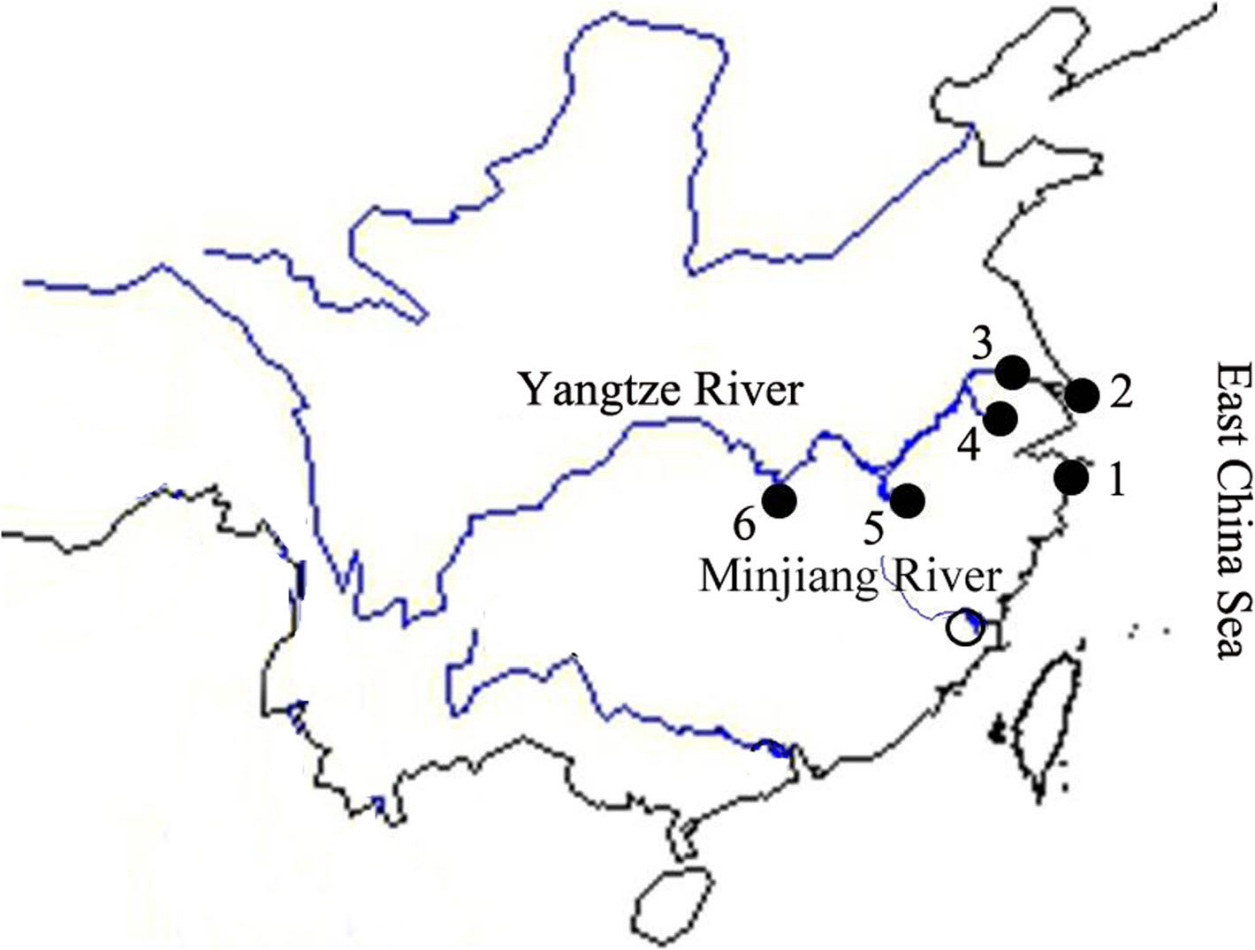
Sampling locations used for *C. nasus* collection. The numbered dots correspond to the following locations along the Yangtze River: 1, Xiangshan; 2, Chongming; 3, Jingjiang; 4, Taihu Lake; 5, Poyang Lake; and 6, Dongting Lake.

During 2009–2013, we collected approximately 1200 individual samples of *C. nasus* from six locations in China. Of these locations, five were in the middle and lower reaches of the Yangtze River: Chongming (CM), Jingjiang (JJ), Taihu Lake (TH), Poyang Lake (PY), and Dongting Lake (DT). The 6th location was in Xiangshan (XS), which is in the coastal region of the East Sea in China (Fig. 1). Sampling from these six locations ensured the inclusion of all ecotypes of *C. nasus.*

The CM and JJ samples were captured by net when the fish returned home from the sea to freshwater habitats. After the anadromous individuals had left Poyang Lake and Dongting Lake and returned to the sea, resident individuals were still present in the lakes, and samples were caught by net. In order to avoid of using admixture/ hybridization individuals of the two ecotypes as reported in our previous study [33], the representative samples of the anadromous ecotype from CM, JJ and XS populations were chose from the 1200 samples based on the maxilla extending well beyond the gill cover, and the captured time between March to April (Fishes began to migrate from sea back to river). The representative samples of the resident ecotype from PY and DT were selected from the 1200 samples based on the maxilla significantly shorter than the length of the gill cover, and the capture times within January (Fishes are resident in lakes). Ten individual genomes from each population were examined for SINE insertion analysis. The genome of one sample from CM was used to isolate SINE insertion sites. Additionally, three samples of *Coilia mystus* were collected from the Minjiang River in Fujian Province and were used as an outgroup related to *C. nasus*. Muscle tissue for downstream analyses was preserved in 95% ethanol.

### Isolation and identification of SINEs

Genomic DNA enrichment was performed according to the amplified fragment length polymorphism (AFLP) technique as described previously [34]. This step was carried out to isolate and characterize the SINEs in *C. nasus*. The genomic DNA was digested with EcoR1 and BcII (Takara, China), purified, and ligated to either the EcoRI adaptor [34] or the modified MseI adaptor (Additional file 1: Table S1) using T4 DNA ligase (Takara, China). Genomic DNA was amplified by PCR with EcoRI and MseI primers (Additional file 1: Table S1). A total of 5 PCRs for genomic DNA of each individual were performed with 14.5 μL of ddH_2_O, 1.0 μL of 10 mM dNTPs, 2.5 μL of 10× PCR buffer, 0.5 μL of each 10 μM primer, 0.5 μL of Taq, and 0.5 μL of the adaptor-ligated DNA product. The PCR program was as follows: 94 °C for 3 min, followed by 17 cycles of 94 °C for 30 s, 55 °C for 30 s, and 72 °C for 1 min 30 s; and finally, a 10-min extension step at 72 °C. The 5 PCR products were electrophoresed on a 2% agarose gel. Fragments in the 500- to 1000-bp range were eluted using a DNA gel purification kit (Sangon, China).

A magnetic particle system was established using MagneSphere magnetic separation products (Promega, Germany) following the manufacturer’s instructions. The procedure for SINE isolation from the genome of *C. nasus* was described in our previous study [35]. Briefly, the AFLP-enriched DNA fragments were denatured and then hybridized with the 5′-biotin-labeled probe sequence specific to a 120-bp internal region of the SINEs. The biotin present at the 5′ end of the probe specifically attached to the magnetic particles. The probe-target DNA complexes were eluted from the magnetic particles.

The eluted DNA was used as a template for PCR with EcoRI and MseI primers (Additional file 1: Table S1). The PCR products were electrophoresed on a 2.0% agarose gel. Fragments in the 500–1000-bp range were eluted and ligated into the pGMD19-T vector (Takara, China), and the constructs were transformed into *E. coli* DH5α competent cells. Recombinant colonies were screened by PCR with the Sc-F and Sc-R primers (Additional file 1: Table S1), which correspond to the sequence of the conserved region of SINEs. The positive colonies were sequenced using an automated DNA sequencer (ABI PRISM 3730).

Sequences of positive clones were aligned with the primary SINE consensus sequence (Additional file 2: Table S2). Then, the sequences with high scores were searched against zebrafish DNA sources using the RepeatMasker web server (www.repeatmasker.org) to classify the repetitive elements. Finally, sequences that were similar to known repeat elements in Repbase were retrieved via the CENSOR algorithm [36].

### Tails of retrotransposon LINEs and secondary structures

To test whether the new SINEs transpose via a tail region similar to that of retrotransposon LINEs, the genome walking method was used to identify the tail sequence of the LINE using one arbitrary degenerate primer obtained from a kit (Takara, China) and a special primer designed specifically for the LINEs of *C. nasus* [35]. The entire PCR process was conducted according to the manufacturer’s instructions for the kit. The secondary structures of the tail portions of the SINEs and LINEs were predicted using the Mfold tool as previously described [37].

The isolated SINE elements were used to determine the consensus sequence for the primary sequence of the SINE family. To determine the possible tRNA ancestry of SINEs, the tRNA-related regions of the SINEs were used to verify the tRNA-like secondary structure via tRNAscan-SE [38].

### Transcriptomic analysis of SINEs

Transcriptomic analysis was used to determine whether the expression of the new SINEs differed in both ecotypes of *C. nasus*. Total RNA was extracted from olfactory tissues of *C. nasus* using the TRIzol Kit (Invitrogen, USA). cDNA library construction and sequencing, sequence data processing, and de novo assembly for RNA-seq assay were performed as previously described [39]. In brief, individuals with phase III of gonad, similar ages and size were used for transcriptome analysis of ecotypes. One individual of JJ and a mixture with 3 individual of CM were used as the migratory transcriptome samples. A mixture of 3 individuals of PY and 3 individuals of DT was used as the resident transcriptome samples. The contig data were used to construct a local BLAST library. Blastn was used to search for significant hits in the library using the consensus SINE sequence as a query. When a contig matched the query with a high score (> 80), the function of a gene within the matched contig was annotated via Blastx against the GenBank database.

### DNA copy number for SINE analysis

To determine whether the SINEs have undergone natural selection, the genomic copy numbers of the SINEs were determined by real-time PCR. The plasmids with SINE insertions and the genomic DNA of *C. nasus* samples collected from the six locations were prepared as standards and samples for real-time PCR. DNA concentrations were measured with a spectrophotometer, and ten-fold serial dilutions were prepared as templates for real-time PCR in an ABI 7500 instrument (ABI). Ten samples were used, and three replicates were included for each. The real-time PCR program was as follows: 95 °C for 5 min, followed by 40 cycles of 95 °C for 10 s, 55 °C for 20 s and 72 °C for 30 s. The 20 μL PCR mixture included 0.5 μl of each primer (Sc-F and Sc-R) and 10 μl of HRM Master Mix 2× (Qiagen, China). Finally, a melting curve analysis was performed after amplification. Standard curve preparation and data analysis were performed with MJ Opticon Monitor (MJ Research, Waltham, MA). The average genome size of *C. nasus* considered to be 3.534 pg based on a previous report [40] is required to normalize the average size of population genomes. Significance in copy number difference was calculated by ANOVA in SPSS 16.0.

### SINE insertion polymorphisms

During pre-examination, samples collected from the six sampling sites (10 individuals per site) (Fig. 1) were screened to analyze SINE insertion polymorphisms. The primers were designed with Primer Premier 6.0 [41] and were specific for the flanking sequences of each insertion. Finally, five of the 71 total insertion loci (obtained via SINE DNA capture and cloning, as described above) showed polymorphic bands (the PCR primers are listed in Additional file 3: Table S3). PCR was performed for 35 cycles in a 2:l reaction volume using Taq Master Mix 2× (TianGen, China). The allele bands of the five insertion loci were separated, cloned, and sequenced. To confirm the presence/absence of SINE insertions, three to five clones were selected for sequencing per allele at a single locus.

Allele frequencies, gene diversity and exact *p*-value tests for Hardy-Weinberg equilibrium departure were estimated using PowerMarker v.3.25 [42]. Statistical significance for the Hardy-Weinberg equilibrium departure test was considered to be reached at *p* = 0.001. The index for each locus showed the intensity and direction of deviation from the overall value. An AMOVA test was performed to clarify the hierarchical apportioning of SINE frequency variance using Arlequin ver. 3.5 [43]. To analysis genetic relationships among populations, a neighbor-joining (NJ) tree was constructed based on allele frequencies using PowerMarker v.3.25 [42].

The evolutionary history of SINEs in *C. nasus* was evaluated by utilizing *C. mystus* as a comparison outgroup. Three individuals per species were evaluated by PCR with these locus insertion primers. The presence of an expected insertion in *C. mystus* suggests a relatively old insertion in *C. nasus*, and the absence of an expected insertion in *C. mystus* suggests a relatively recent insertion in *C. nasus*.

## Results

### Molecular characterization of SINEs

**Fig. 2:**
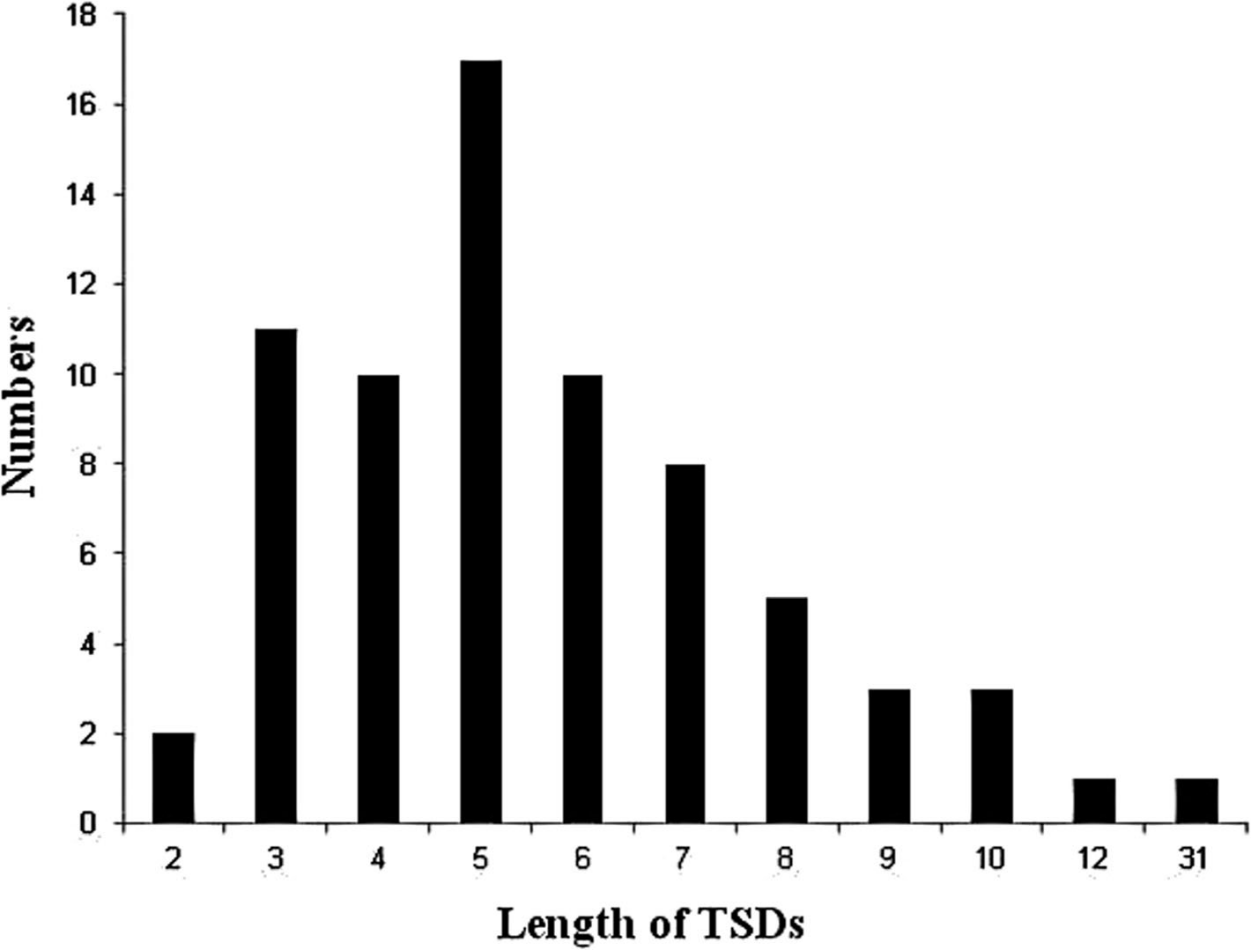
Length distribution of target site duplications (TSDs) in the SINE insertion flanking regions of *C. nasus*

**Fig. 3:**
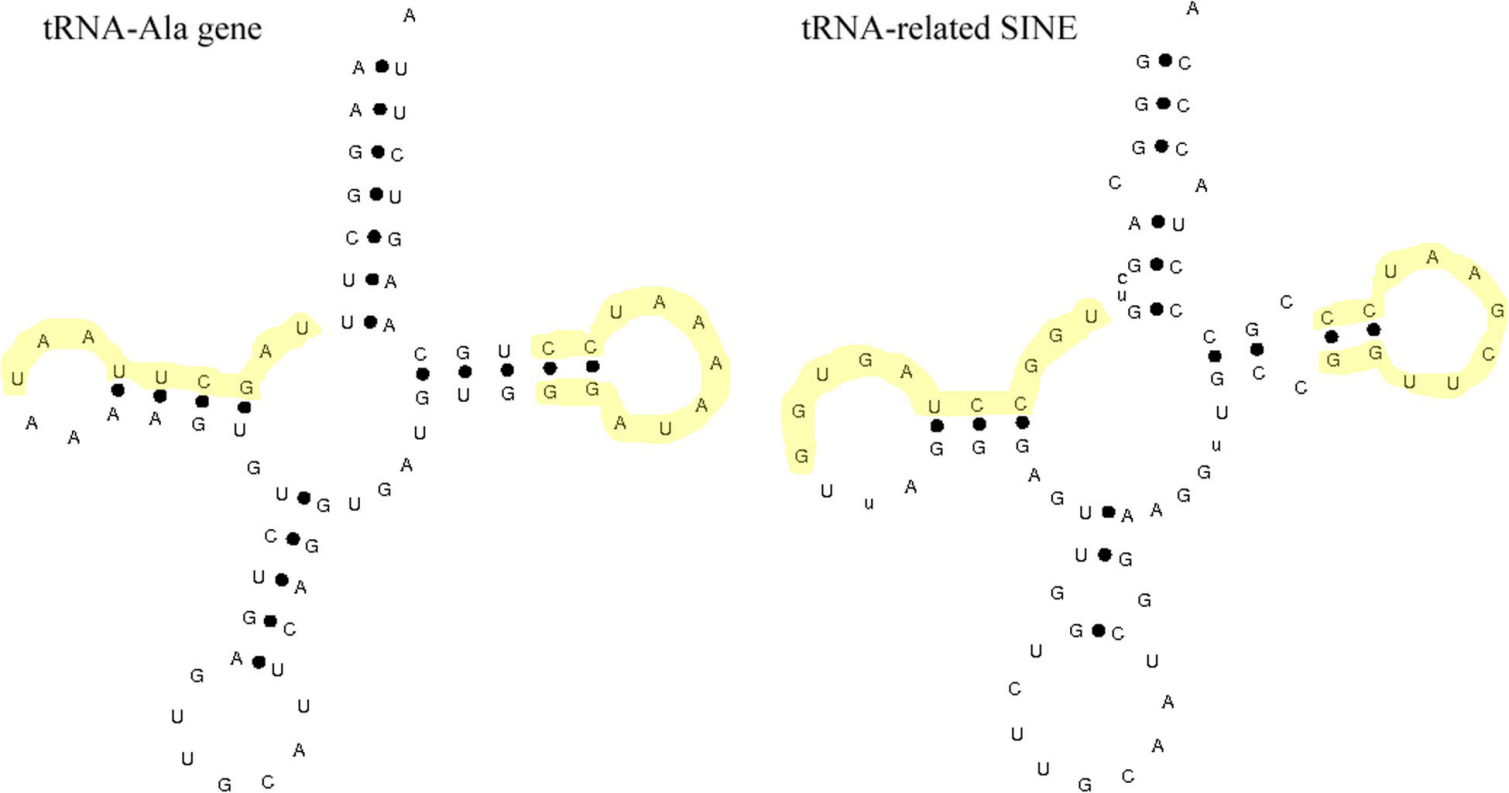
Cloverleaf structures of the tRNA-related SINEs in *C. nasus* and the tRNA^Ala^ (UGC) gene. The highlighted yellow region denotes the A and B boxes in the tRNA-related SINE as defined previously (1).

A total of 259 SINE clones were identified in the *C. nasus* genome. After sequencing, redundant sequences were deleted. The resulting sequences were aligned with the SINE consensus sequence. We obtained 71 validated positive clones with SINEs. These SINEs vary between 178 and 285 bp in size and contain the flanking sequences of the insertion locus, which have target site duplications (TSDs) ranging from 2 to 31 nucleotides, although a majority of the TSDs range in size between 3 and 8 nucleotides (Fig. 2). These SINE clones are 77–94% similar to the SINE consensus sequence, confirming that these clones belong to the SINE family. The sequences of the SINEs in the *C. nasus* genome showed that the tRNA-related regions of the SINEs, which are approximately 75 bp in length, originated from six ancient tRNA genes (Additional file 4: Table S4) and can be folded into perfect cloverleaf tRNA structures, despite low similarity (49%) between their primary sequences (Fig. 3). Overall, our results indicate that these SINEs originated from ancient tRNA genes.

The body regions of SINE elements vary in length from 60 to 170 bp because of either a 41–91-bp insertion or 3–31 bp indels (insertions and deletions). We found that the deletions occurred randomly, but the insertions occurred largely in the nuclear repeat regions. The 3′ tail regions of the SINE elements exhibited 76–99% conservation and contained a 56-bp tail block similar to that of L2–2-DRe, a LINE retrotransposon from the zebrafish genome [44]. Considering the importance of the conserved poly(A) or TGTAA motif in the 3′ tail region of SINE transposons [14], we searched for these motifs in SINEs with 3′ tails similar to those of LINEs. Three motif patterns, poly(A), TGTAA, and TGTAA-poly(A), were identified.

**Fig. 4:**
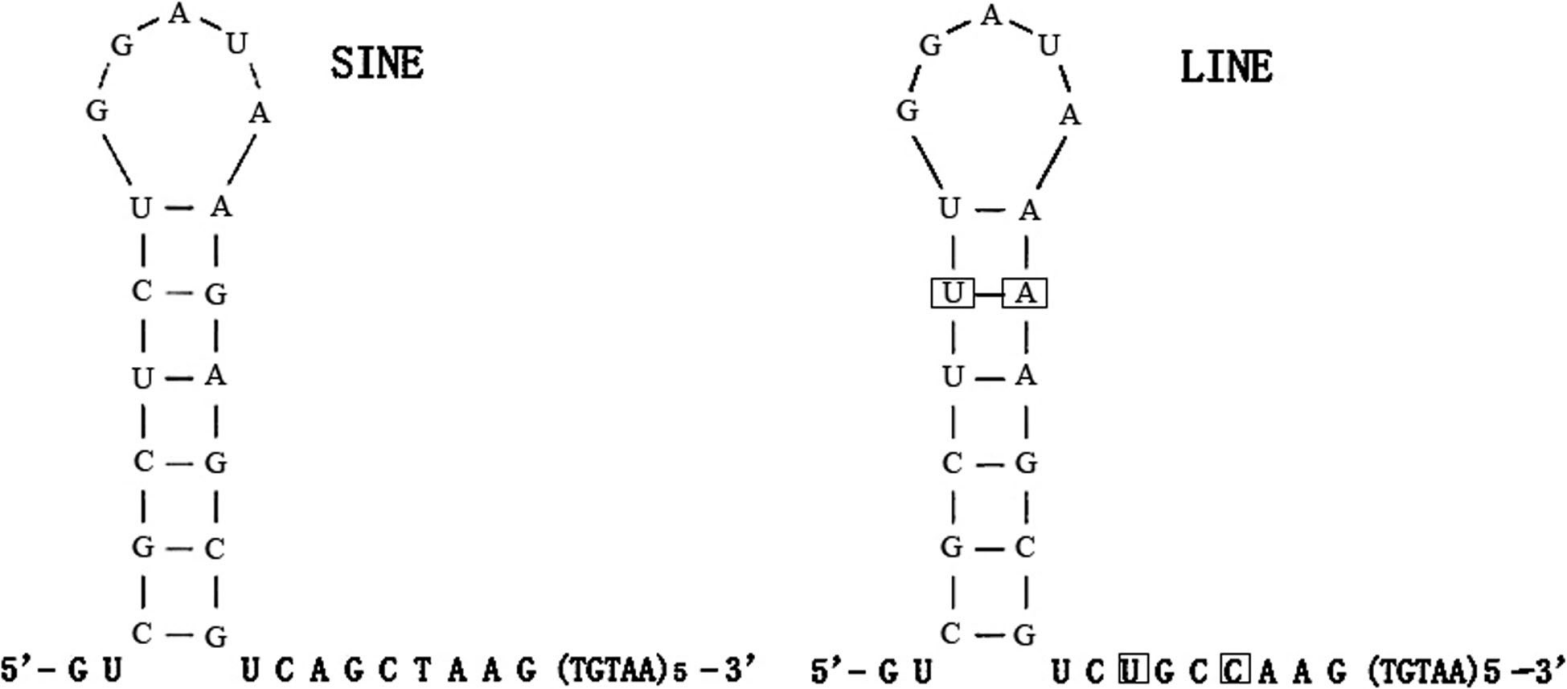
Secondary structures of the tail regions of the SINEs and LINEs in *C. nasus*. The boxed positions represent nucleotides that differ between SINEs and LINEs

The transposition ability of these SINEs in the *C. nasus* genome depends on whether the organism shares these motif patterns in the tails of its LINEs. Interestingly, the 3′ tails of LINEs from *C. nasus* range from 337 to 402 bp and exhibit 58–64% similarity. This low similarity is due to the variation in the 3′ region of the LINEs; however, this region harbors two types of motifs, namely, poly(A) and TGTAA motifs. Both motifs exhibited high similarity with the 53-bp tail of the SINEs and shared a stem-loop secondary structure and five TGTAA repeats (Fig. 4). The results supported that the nonautonomous SINEs can mobilize via both the slippage reaction and recognition by the LINE reverse transposase [15].

### SINE transcription differs between ecotypic populations

**Fig. 5:**
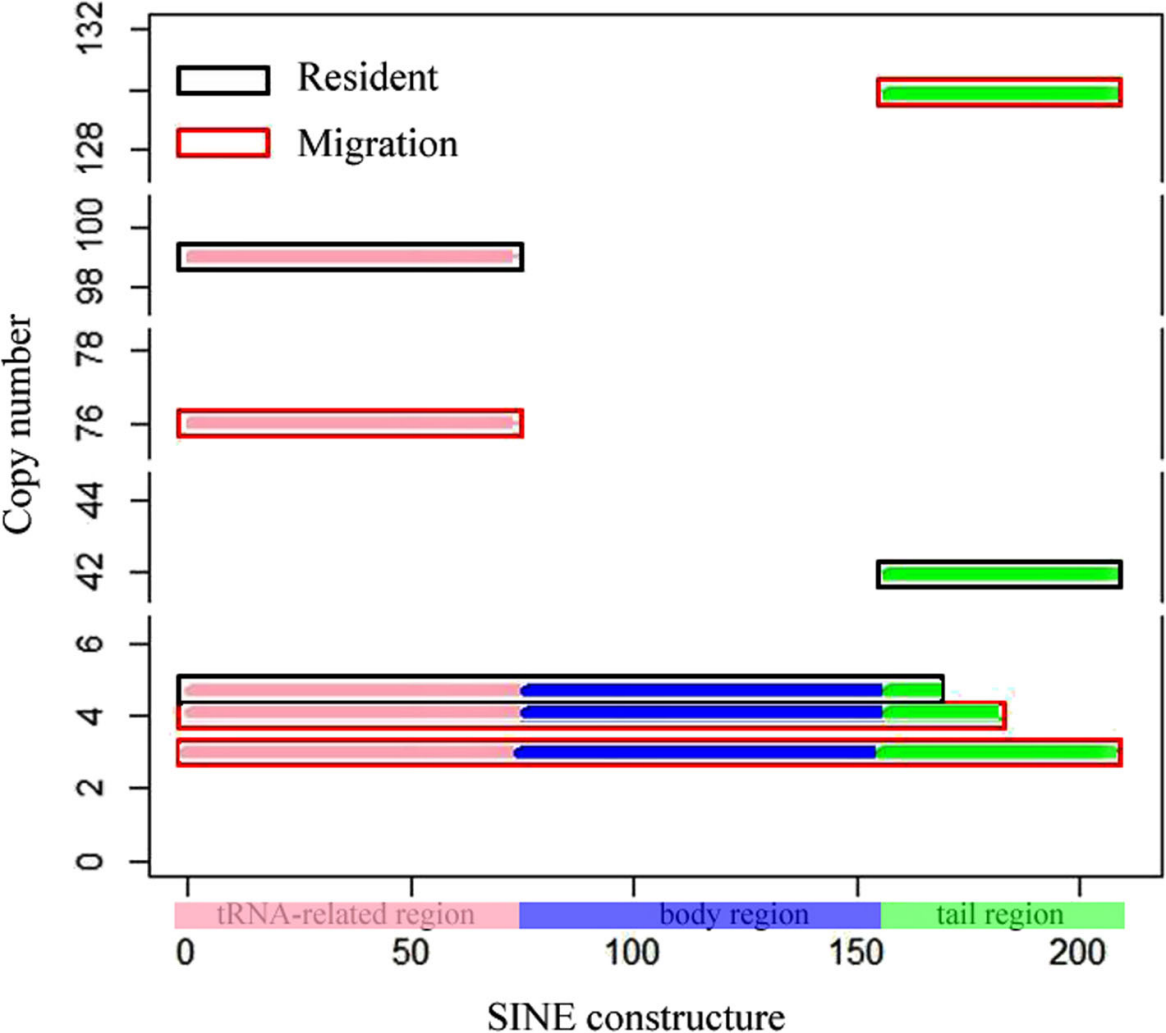
RNA copy numbers of SINEs in two ecotypes of *C. nasus.* The diagram of the 208-bp SINE at the bottom is stratified into three regions: the tRNA-related region (75 bp, shown in pink), the body region (shown in blue) and the tail region (56 bp, shown in green)

To investigate whether the RNA intermediates of SINEs that involve retrotransposition are transcribed at the replicative stage of mobilization, two transcriptomes of olfactory tissues from the migratory and resident types were constructed by de novo assembly. A data set of migratory transcriptome comprised of one individual of JJ (NCBI SRA: SRP035517) and a mixture with 3 individual of CM (NCBI SRA: SRP100816). The other data set of resident transcriptome consisted of a mixture with 3 individual of PY (SRP035517) and a mixture with 3 individual of DT (SRP100816). With the two data sets, 343,265 and 491,297 contigs were obtained from the combined reads in the transcriptomes of two ecotypes, migratory type and resident type, respectively. Using the SINE consensus sequence as a query, we obtained significant hits as SINE copy numbers from the two sets of contig data. Three complete transcript copies of the SINEs were found in the migratory type but not in the resident type (Fig. 5). A complete SINE element has three regions: a tRNA-related region (75 bp), a body region (75 bp) and a 3′ tail region (56 bp) (Fig. 5). The copy number of the tRNA-related region in the migratory type was slightly lower than that in the resident type. The copy number of the 3′ tail region in the migratory type was significantly higher than that in the resident type (Fig. 5). The observed difference in SINE expression in these two *C. nasus* ecotypes may explain their genetic variation and species differentiation.

Out of the total number of contig hits with SINEs, 42 of the resident type and 34 of the migratory type, contain genes with annotated functions related to signal transduction, cell structure, and transporter activity (Additional file 5 and Additional file 6: Tables S5 and S6). Importantly, two genes within the SINE-hit contigs were found to be known genes that are important for migrating *C. nasus* fish, namely, the genes encoding the S100 calcium-binding protein and the interferon regulatory factor. These genes that exhibited differences between the two transcriptomes may explain the different life histories of *C. nasus*.

### SINEs underwent strong natural selection that resulted in genomic disparity between populations

Given our finding that the complete RNA transcripts of SINEs were present in the migratory type but not in the resident type, we next focused on examining whether SINEs had undergone natural selection in the resident type via DNA copy number analysis of SINEs. The DNA copy numbers of the SINEs were quantified by real-time PCR. The DNA copy number in each sample was calculated by generating a standard curve between the cycle threshold (Ct) and the log of the initial template copy number using the regression equation y = − 3.11 x + 40.838 (*R*^2^ = 0.999).

**Fig. 6:**
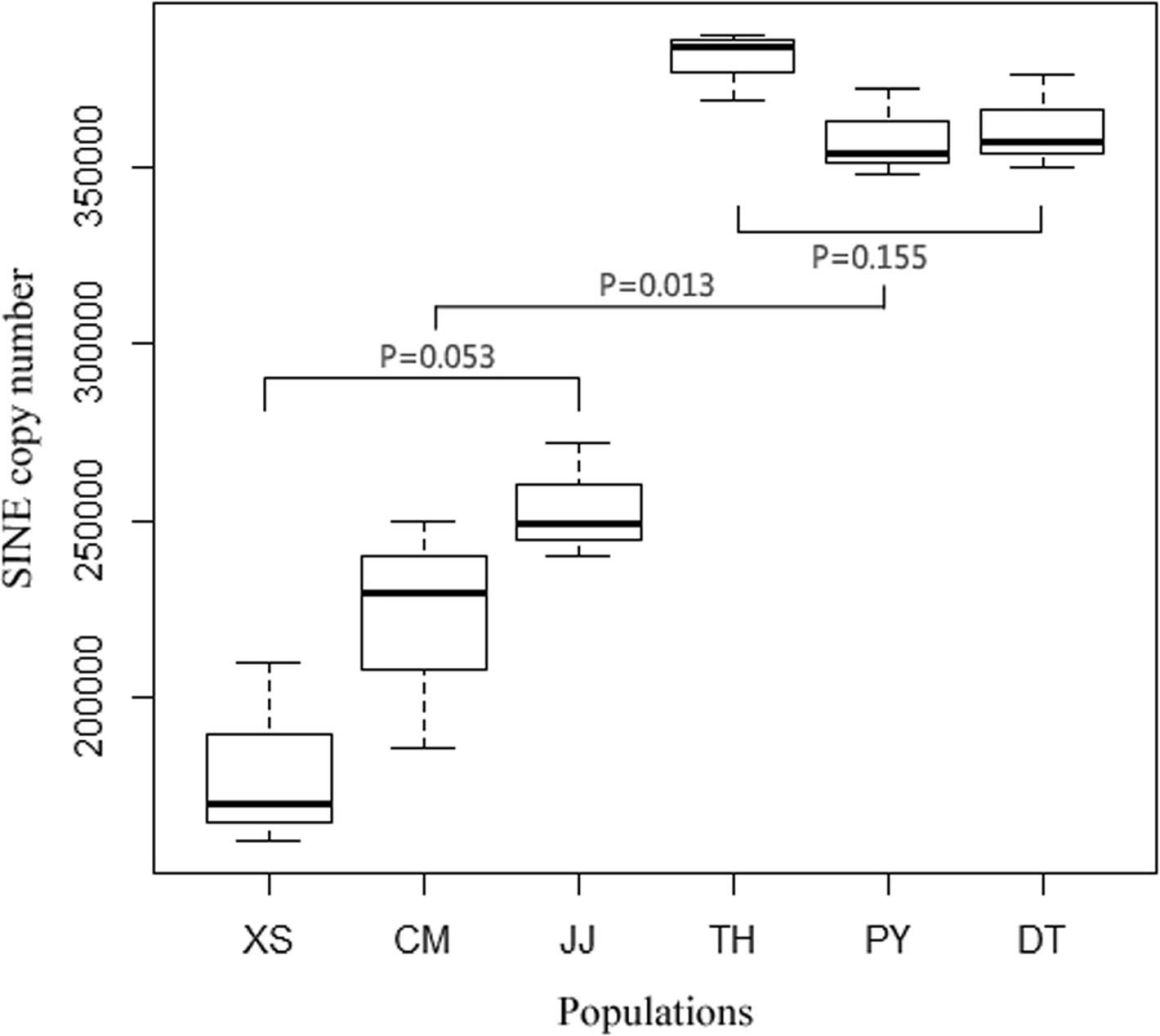
SINE DNA copy number estimates in the genomes of the *C. nasus* populations. Abbreviation: XS, Xiangshan; CM, Chongming; JJ, Jingjiang; TH, Taihu Lake; PY, Poyang Lake; DT, Dongting Lake. P denotes a significant difference. The migratory ecotype includes XS, CM and JJ, while the resident ecotype includes TH, PY and DT

The average DNA copy numbers from the six sampling sites varied between 1.8 × 10^5^ in XS to 3.8 × 10^5^ in TH (Fig. 6). The average DNA copy number was 3.5 × 10^5^ in the resident type and 2.3 × 10^5^ in the migratory type. The DNA copy numbers of the SINEs showed no significant difference within the ecotypes (*p* > 0.05) but a significant difference between the ecotypes (*p* <  0.05).

**Fig. 7:**
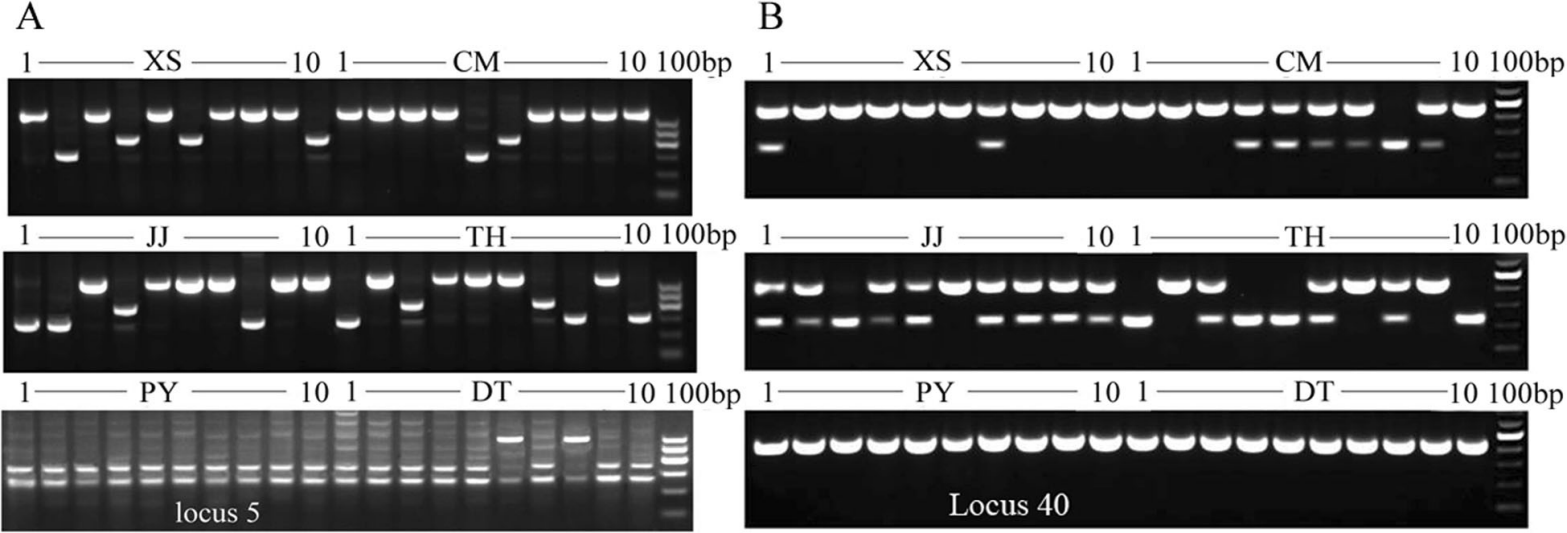
SINE insertion patterns in populations of *C. nasus* at two representative loci. **a** denotes locus Ls5, **b** denotes locus Ls40, XS denotes Xiangshan samples, CM denotes Chongming samples, JJ denotes Jingjiang samples, TH denotes Taihu Lake samples, PY denotes Poyang Lake samples, and DT denotes Dongting Lake samples

We next examined the SINE insertion polymorphisms among the ecological populations. Five loci, namely, the Ls5, Ls29, Ls40, Ls58 and Ls60 loci, from 71 validated positive clones, were found to have insertion polymorphisms in the two *C. nasus* ecotypes via PCR. For Ls5, three bands were observed in all the samples from the six populations (Fig. 7a). By sequencing verification, we found that the largest band (644 bp) contained an insertion, the second band (358 bp) was an insertion-free fragment, and the smallest band (282 bp) represented a nonspecific PCR amplification product. The Ls40 locus was polymorphic, showing the presence or absence of a SINE insertion in all samples (Fig. 7b). The polymorphisms at loci Ls29, Ls58 and Ls60, which were caused by the presence/absence of insertions, were also confirmed via a similar cloning and sequencing procedure.

We also found that the heterozygosity of the SINE insertions—a value that indicates the genetic diversity—was low in all the samples. The heterozygosity value was zero for the Ls5, Ls29 and Ls58 loci and slightly higher than 0 for the Ls40 and Ls60 loci in all six populations (Table 1). This result demonstrates a low heterozygosity level in the *C. nasus* genome. Next, we compared the SINE heterozygosity between the two *C. nasus* ecotypes. Although the heterozygosity value was not significantly different within ecotypes, the value for the migratory type was ~ 1.5-fold higher than that of the resident type across all five loci, and three loci (Ls5, Ls29 and Ls58) deviated significantly from Hardy-Weinberg equilibrium (Table 2). These results suggest the existence of a disparity distribution of SINE insertions in the *C. nasus* genome due to natural selection.

**Table 1:** Allele frequencies and heterozygosities of SINE insertions in *C. nasus* populations

Locus	XS		CM		JJ		TH		PY		DT		Total Pop	
	Het	ƒ_SINE_	Het	ƒ_SINE_	Het	ƒ_SINE_	Het	ƒ_SINE_	Het	ƒ_SINE_	Het	ƒ_SINE_	ƒ_SINE_	Het
Ls40	0.90	0.20	0.65	0.50	0.50	0.80	0.45	0.30	1.00	0.00	1.00	0.00	0.75	0.30
Ls5	0.60	0.00	0.80	0.00	0.60	0.00	0.50	0.00	0.00	0.00	0.20	0.00	0.45	0.00
Ls58	1.00	0.00	1.00	0.00	1.00	0.00	1.00	0.00	0.10	0.00	0.00	0.00	0.68	0.00
Ls60	0.00	0.00	0.00	0.00	0.00	0.00	0.00	0.00	0.50	1.00	0.50	1.00	0.17	0.33
Ls29	1.00	0.00	1.00	0.00	1.00	0.00	1.00	0.00	0.00	0.00	0.00	0.00	0.67	0.00
Mean	0.70	0.04	0.69	0.01	0.62	0.16	0.59	0.06	0.32	0.20	0.34	0.20	0.54	0.13

Abbreviations: *XS* Xiangshan; *CM* Chongming; *JJ* Jingjiang; *TH* Taihu Lake; *PY* Poyang Lake; *DT* Dongting Lake. The f_sine_ values donate SINE frequency, and Het donates genomic heterozygosity

**Table 2:** Allele frequencies and heterozygosity values of five SINE insertion loci in *C. nasus* ecotypes. The f_sine_ values denote SINE frequency, Het denotes genomic heterozygosity, and *P*-value denotes the statistical significance of the Hardy-Weinberg equilibrium departure test

Locus	Migration		Residence		*P* -value
	ƒ_SINE_	Het	ƒ_SINE_	Het	
Ls40	0.57	0.65	1.00	0.00	0.855
Ls5	0.70	0.00	0.10	0.00	< 0.001
Ls58	1.00	0.00	0.05	0.00	< 0.001
Ls60	0.00	0.00	0.50	1.00	0.035
Ls29	1.00	0.00	0.00	0.00	< 0.001
Mean	0.65	0.13	0.33	0.20	

**Fig. 8:**
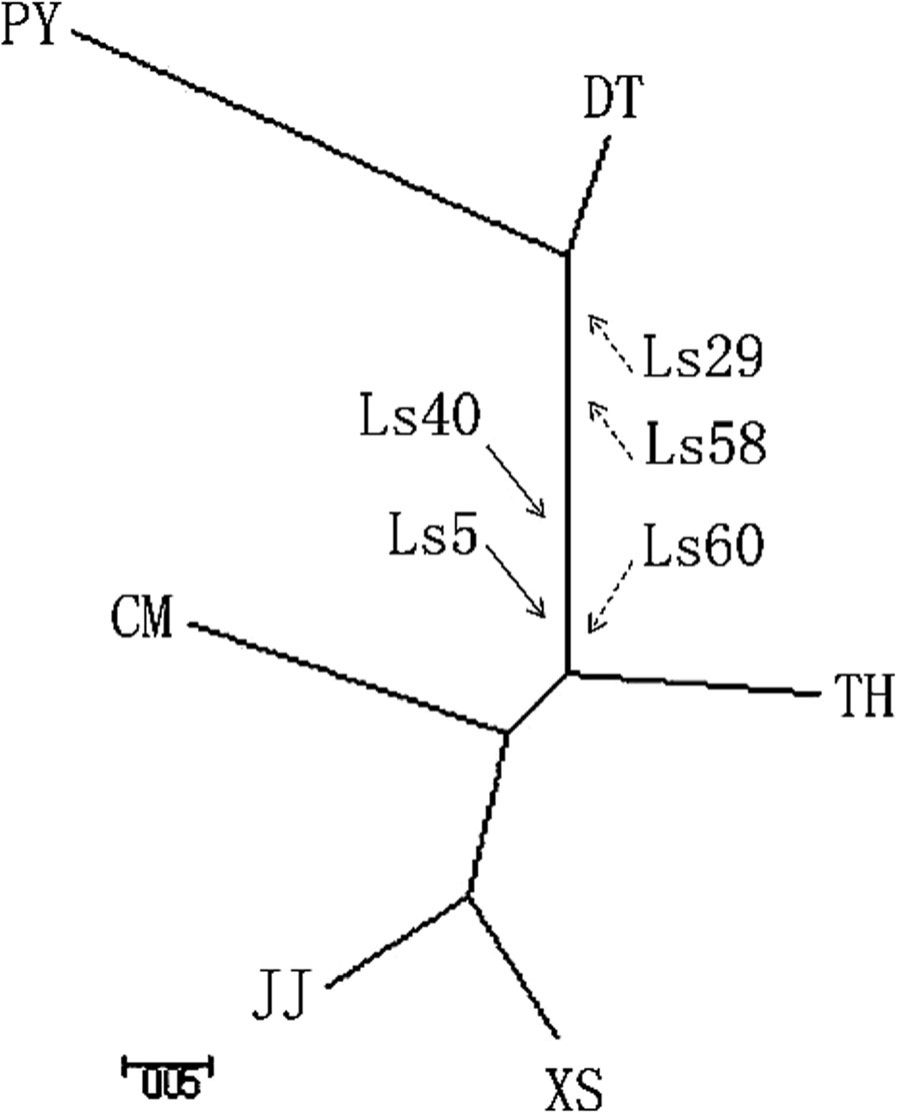
Neighbor-joining tree illustrating population differentiation in *C. nasus* Abbreviations: XS, Xiangshan; CM, Chongming; JJ, Jingjiang; TH, Taihu Lake; PY, Poyang Lake; DT, Dongting Lake. Dashed arrows indicate the absence of SINE insertion; solid arrows indicate the presence of SINE insertion.

To examine the differentiation between these populations, a neighbor-joining (NJ) tree was constructed based on the allele frequencies of SINE insertion (Table 1). The NJ tree clearly illustrated a lineage of two ecotypes. XS, JJ, CM and TH grouped as the migratory type, while DT and PY grouped as the resident type. Of the migratory populations, TH was most closely related to the resident ecotype (Fig. 8). Although the migratory type displayed genetic variation, populations of the migratory type (TH excluded) were to compare the genetic variance of TH population, and no difference was observed between populations (*P* > 0.05), suggesting no significantly genetic differentiation between the migratory type (TH excluded) and TH population. In contrast, the genetic variance in populations of the resident type compared with TH population was 75–25% (*P* <  0.5). Therefore, this implies that there is genetic diversification between migratory and resident *C. nasus* in the Yangtze River. The lack of SINEs in the resident type (Ls29 and Ls58) and migratory type (Ls60), together with the SINE insertions in the migratory type (Fig. 8), may have contributed to the genetic diversity of *C. nasus*. Thus our findings may assist in the examination of the life history diversity of *C. nasus*.

### SINE excision revealed microhomology-induced replication

SINE insertions that underwent natural selection led to a disparity distribution in these populations. To determine the evolutionary scale of SINE insertions, including those at the Ls5, Ls29, Ls40, Ls58 and Ls60 loci, we utilized *C. mystus*, a species that is closely related to *C. nasus*, as an outgroup for comparison. We found insertions at the Ls29, Ls58 and Ls60 loci in both species. Insertions at the Ls5 and Ls40 loci were present in *C. nasus* but absent in *C. mystus*, suggesting that these insertions originated from a recent transposition activity in *C. nasus*, and the Ls29 and Ls58 insertions, which were absent in the resident type of *C. nasus*, had undergone excision.

**Fig. 9:**
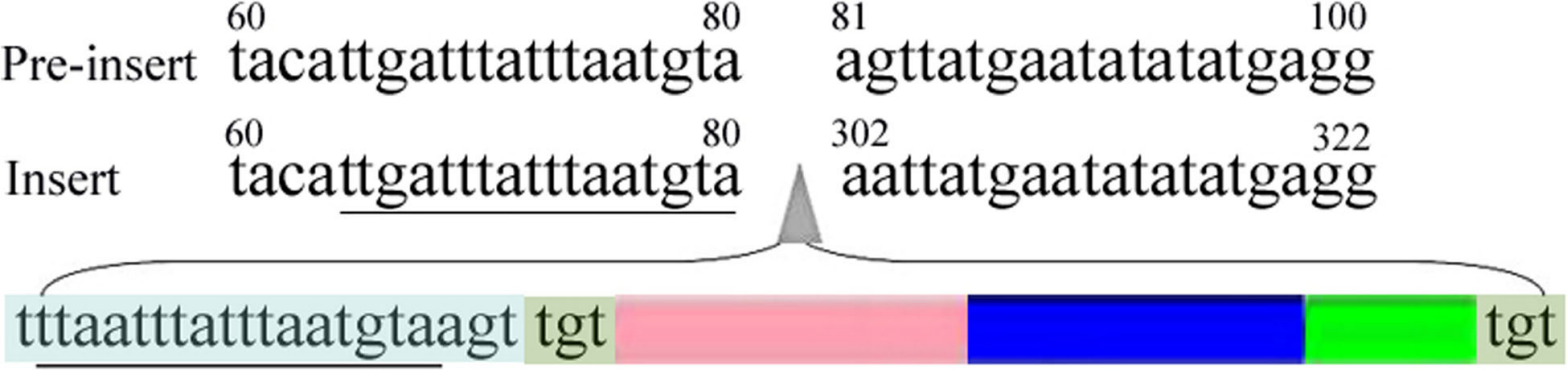
Diagram of repeat motifs resulting from SINE insertion in *C. nasus*. The upper mode denotes a preinserted locus and the absence of the SINE in *C. mystus*, which was used as an outgroup. The lower mode denotes a SINE insertion in the resident type of *C. nasus*. The “tgt” denotes TSDs at both ends of the SINE insertion. The block in pink-blue-green denotes a SINE element with three parts: tRNA-related region, body region and tail region.

**Fig. 10:**
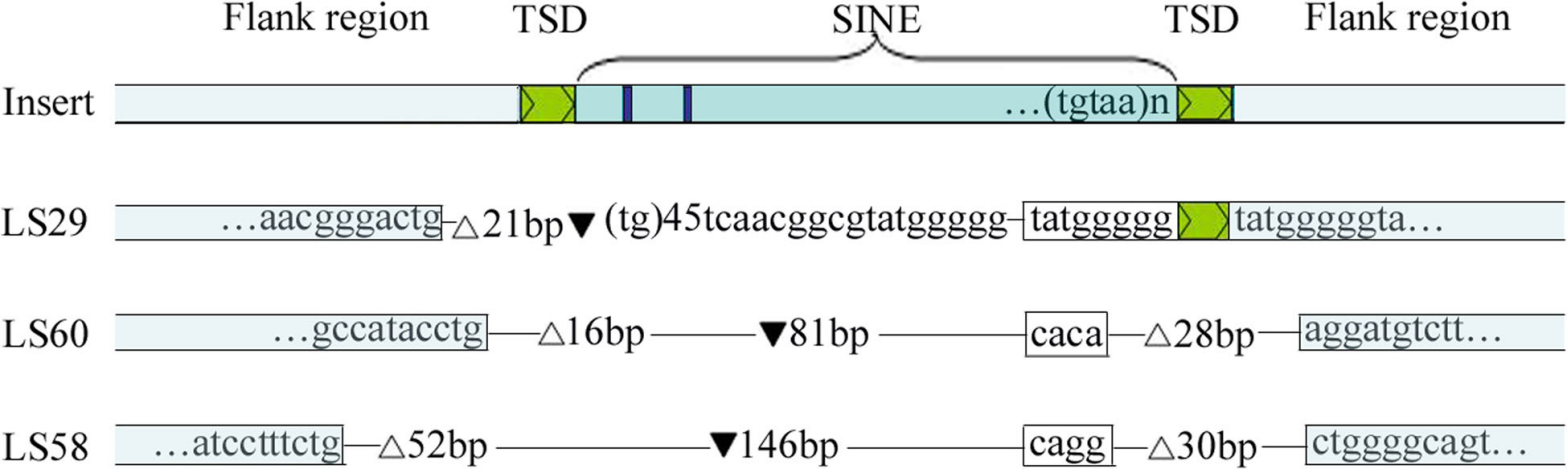
Diagram of SINE excision events in *C. nasus*. The upper portion denotes the pattern of SINE insertions in the migratory type, and the lower portion denotes the pattern of SINE excision in the resident type. The white triangle denotes excised nucleotides, and the black triangle denotes the inserted nucleotides at the excision sites. The white boxed sequences exhibit microhomologies with the insertion sequences.

To test whether the SINE-specific burst in *C. nasus* had occurred recently, we sequenced the flanking region of the Ls5 locus; a “TGT” TSD was observed at the terminus of the insertion, and a 17-bp duplication was observed at the preinsertion sites at the 5′ terminal flanking region of the insertion site (Fig. 9). Our results provide evidence of SINE insertion in *C. nasus* because TSDs are present at these loci. For SINE insertion-excision, we expected footprints that would be present in the corresponding insertion-lacking locus. We observed incomplete SINE excision at the Ls29, Ls58 and Ls60 loci. For Ls29, the SINE 5′-flanking portion was excised. The gap was filled with a fragment (up to 115 bp) consisting of a microsatellite of 45 (TG) repeats and an 8-bp microhomology domain with the SINE 3′ terminal flanking sequences (Fig. 10). For Ls58 and Ls60, various deletions in the flanking sequences and short microhomologies in the repaired DNA were observed (Fig. 10). Our findings support the idea that chromosomal breakpoints can be joined via microhomology-induced replication and that double-stranded break repair followed by template switching between microhomologous sequences can lead to the generation of a new sequence to refill the excision site [45].

This observation suggested that if mutations occurred in the flanking sequences of a SINE insertion locus, a new cleavage site could be formed, and the insertion could be incompletely excised, and followed by sequence changes from DNA repair. A region with a repeat motif should be prone to mutations, as observed in the 3′ tails of the SINEs via transcriptomic analysis. Upon examining whether the 5′ flanking regions of the SINEs have repeat motifs at nearby insertion loci, we observed that the 50-bp sequences immediately upstream of the SINE 5′ flanking regions were enriched in A and T residues (mean = 56.6%). The AT content was significantly higher than the GC content (*P* <  0.05). The 50-bp sequences immediately downstream of the SINE 3′ flanking regions were similarly AT rich (mean = 57%). However, tandem repeats were predominantly found in the upstream region. These tandem repeats, which were up to 100 bp in length, were found at 13 loci. In contrast, only one locus was found to have a tandem repeat located in the downstream region (Table 3). The properties of the repeats in the 5′ flanking and 3′ tail sequences of SINEs provide crucial clues regarding the mechanism of SINE insertion-excision and replication by the organism.

**Table 3:** Characteristics of the simple repeats in the SINE flanking regions

Loci	Flanks	Repeat unit	Sites
Ls2	5′	(G)38	−76
Ls61	5’	(A)17	−1
Ls11	5’	(TG)26	−55
Ls26	5’	(CA)16	−56
Ls69	5’	(CA)14	−48
Ls29	5’	(CG)18	−16
Ls62	5’	(CT)7	−85
Ls52	5’	(AAC)17(AAT)8	−3
Ls32	5’	(ATTAC)7	−2
Ls18	5’	(TTATTTAA)3	−10
Ls12	5’	(TTGGACCAGC)6	−3
Ls43	5’	(ACTAGGGAACTACCAGGGGG)14	−8
Ls44	3’	(GT)10	+ 115

## Discussion

### A novel SINE family isolated from *C. nasus*

In this study, we isolated new SINE family members from *C. nasus*. Based on GenBank or Repbase database searches, the 208-bp consensus sequence of the new SINE members showed no hits from other organisms [36]. However, in another search, these SINEs were identified in 19 species of the family Engraulidae (data not shown). Thus, this type of retrotransposon was first identified in this family.

These SINEs are conserved in the tRNA-related region and can fold to form a perfect cloverleaf tRNA structure, which is important for the initiation of SINE RNA transcription (Fig. 3) [15]. Generally, the SINE tRNA-related region is followed by a strongly conserved region called the body region, which was previously thought to represent a common origin in the same species [2]. However, our results showed that the body regions of the *C. nasus* SINEs share < 50% sequence identity, a value that is less than the defined threshold (76%) of the conserved regions in the same SINE family [46–49]. Therefore, the body regions of the SINEs identified in this study are not homologous to those of the existing SINE families, suggesting a different origin from those of the known SINEs.

### Expression divergence of SINEs in *C. nasus* populations

To determine whether the transcript copy numbers of SINEs differ between the two ecotypes of *C. nasus* at the RNA level, we compared the transcriptomic profiles of the two ecotypes. The migratory type contains more SINE contig hits than the resident type (Fig. 5). Most of the hit contigs showed variations due to nucleotide substitution, insertions and deletions. The sequence divergence of SINEs as mobile elements, as previously reported, can be targeted by uniquely mapping short interfering RNAs (siRNAs), which are involved in efficient siRNA-mediated methylation at the mobile elements [50]. Subsequently, the methylated mobile element can strongly regulate the expression of adjacent genes [50–52], leading to further species divergence [53, 54]. Therefore, it is reasonable to propose a significant role for SINEs in the ecotypic divergence of *C. nasus*.

In this study, the effects of SINEs on gene expression were also observed in two transcripts associated with SINE insertions. The first transcript was the interferon regulatory factor gene in the resident type, which contains a SINE insertion in its 3′ downstream region; this gene is known to respond to viral infection [55]. The second transcript was the S100 gene in the migratory type, which is involved in the regulation of cytosolic calcium concentration during migration from the ocean to fresh water [56]. Our results suggest that SINEs make important contribution to gene expression variation in *C. nasus* and likely influence the differences in life history of this species.

### Implications of the skewed frequency and copy number for purifying selection

Purifying selection in evolution leads to a transposition selection equilibrium that occurs via elimination of deleterious insertions to limit population frequencies [57]. A disparity insertion indicates an adaptive event [18]. In this study, we found disparity SINE insertions among the *C. nasus* populations sampled from six sites, and some insertions were associated with life history factors in *C. nasus*. At the population level, the mean insertion frequency of SINEs in three populations of the migratory type (0.62–0.7) was higher than that in the two populations with resident behavior (0.32–0.34, see Table 1). Similarly, the mean insertion frequency of the migratory type (0.65) was higher than that of the resident type (0.33, see Table 2). This disparity distribution of insertion frequency suggested that the SINE sites have undergone purifying selection to further polarize the populations [58].

It is plausible that these SINE insertions purified via natural selection have impacted on the differences in life history of the *C. nasus* ecotypes. *C. nasus* initially originated in the ocean and migrated into rivers and lakes for reproduction [59]. Therefore, this species faced environmental challenges during the reproductive process. However, SINEs can undergo transposition bursts in the genome, allowing *C. nasus* to adapt to the environment via SINE insertions that disrupt gene expression [9]. Even insertions that are separated by large distances in the genome (> 2000 bp) can lead to disruption of promoter-enhancer interactions [50]. These retrotranspositions can be instrumental in increasing the frequency of beneficial insertions into the genome and decreasing deleterious insertions to avoid insertion-related damage [5]. As a result, populations that have achieved beneficial insertions exhibit high SINE copy numbers, especially small populations [19]. In fact, the resident type was a small population relative to the migratory type in terms of fishery catches in the 1950s. The SINE copy number in the *C. nasus* resident type was higher than that in the migratory type (3.5 × 10^5^ vs 2.3 × 10^5^, see also Fig. 6). This result is consistent with results from other studies that have shown how copies of mobile elements have contributed to genomic divergence during population expansions [29, 30, 60].

Phylogenetic analyses of *C. nasus* populations based on SINE insertion frequency readily distinguished two ecotypes of *C. nasus* (Fig. 8)*.* The DT and PY populations were defined as the resident types and were sorted into one group. The XS, JJ and CM populations were sorted into the other group, which was regarded as the migratory type in previous studies [30]. The TH population, which was identified as a subspecies of *C. nasus* in previous studies [59], displayed diversification from both the migratory and resident ecotypes. This result is consistent with our prior study, which showed that the genomic disparity of *C. nasus* was based on polymorphisms at three insertion sites [61]. Taken together, these results show that *C. nasus* can be distinguished into two ecotypes based on the insertion frequency and copy number of SINEs. Our results, together with mitochondrial DNA [29], nuclear DNA and AFLP [62], could help us to clarify the evolutionary history of *C. nasus*.

### Genetic polymorphism driven by SINEs

SINEs can retrotranspose in mammalian genomes because these elements have a 3′ tail structure similar to that of LINE sequences [14, 63]. We observed that the *C. nasus* SINEs and LINEs shared the stem-loop structure of the 3′ tail region (Fig. 4). As in other organisms [15], it is plausible that this stem-loop structure functions as a recognition site for the retrotransposase proteins encoded by LINEs. Retrotransposition of SINEs would have resulted in TSDs at the insertion site via the copy-and-paste mechanism [2]. The SINEs in *C. nasus* showed perfect TSDs at some insertion sites, and a few TSDs were composed of simple repeats in the insertion flanking regions of the SINEs (Table 3). Such repeats are regarded as generators of microsatellites [64]. About 23% of total minisatellites/satellites are derived from transposons in human genome [65].

Although SINE insertion can increase SINE copy numbers in genomes, this process is restricted by various mechanisms in the genome, such as insertion/deletion, genetic drift, and ectopic recombination [17–19, 58]. In this study, we observed incomplete excision of SINEs in *C. nasus* (Fig. 10). Deletion of these SINEs may have given rise to the genetic diversity of this species and led to the further development of a different population via an insertion-selection process, as suggested by others [8, 66].

However, the deletion mechanism has not been explained to date, with the exception of element mutations. In this regard, the SINE evolutionary events that occurred in *C. nasus* may help elucidate the mechanism underlying SINE insertion/deletion. First, the TGTAA short repeat in the SINE 3′ tail could permit template slippage during initiation of DNA replication [14] and generate various numbers of the short repeat in DNA fragments containing SINEs. Second, replication slippage mispairing can increase the rate of mutation [67], which could be used to generate possible cleavage sites for SINEs. Therefore, the SINE element could be incompletely excised, and the gap at the cleavage site could be replicated by the organism. This SINE-based mechanism can be used to explain genetic polymorphism in *C. nasus*.

## Conclusions

In conclusion, novel SINEs were isolated from an anadromous fish, *C. nasus*, which includes a freshwater-resident ecotype. The two ecotypes of *C. nasus* differ in their reproductive behavior. These SINEs are active and underwent a transposition burst in the genome of the anadromous ecotype, producing polymorphic insertions and further influencing gene expression and function. As part of life history adaptation to the freshwater environment, incomplete excisions occurred at a small spatial scale in individuals with habitat specialization, and the benefit conferred by SINE insertions resulted in greater SINE copy numbers in the resident ecotype, eventually facilitating population divergence and speciation. Therefore, SINE activity and incompletely excision led to the ecotypic diversity of the *C. nasus* populations. Our results provide a valuable clue to understanding speciation and population structure within commercially important species.

## Supplementary information

**Additional file 1: Table S1.** Adaptors used to structure the AFLP library and primer sequences used to scan SINE insertions in the library.

**Additional file 2: Table S2.** The consensus sequence of SINE family from genome of *C. nasus.*

**Additional file 3: Table S3.** Primers specific to the flanking sequences of the SINE insertion loci used to detect insertion polymorphisms in *C. nasus* populations.

**Additional file 4: Table S4.** Types, origins and percent similarities of the tRNA-related portions of SINEs in *C. nasus*.

**Additional file 5 Table S5.** Genes annotated in SINE-hit contigs from the migratory type.

**Additional file 6 Table S6.** Genes annotated in SINE-hit contigs from the resident type.

## Data Availability

Sequencing data have been deposited in the NCBI database under the following accession numbers: MK621683–753, SINE elements at the inserted loci; MK621754–757, tail portions of LINE elements; and MK621758–769, sequences of the different bands at each insertion locus.
