# Supplementary material for: SINE Retrotransposon variation drives Ecotypic disparity in natural populations of Coilia nasus
Source: Mob DNA. 2020 Jan 8;11:4. doi: 10.1186/s13100-019-0198-8 (PMC6951006; doi:10.1186/s13100-019-0198-8)
Supplement: Supplementary file 1 — Additional file 1: Table S1. Adaptors used to structure the AFLP library and primer sequences used to scan SINE insertions in the library. [file 13100_2019_198_MOESM1_ESM.pdf]

Supple Table 1 Adapters were used to structure the AFLP library and primer sequence scanned SINE insertions in the library.

EcoRI-adapter top is 5’-CTCGTAGACTGCGTACC-3’

EcoRI-adapter bottom is 5’-AATTGGTACGCAGTCTAC-3’

MseI-adapter top is 5-GACGATGAGTCCTGAG-3

MseI-adapter bottom is 5-GATCCTCAGGAC TCAT-3

EcoRI primer is 5-GACTGCGTACCAATT C-3

Msel primer is 5-GATGAGTCCTGAGGAT-3

Sc-F primer is 5-TAGTGGTTAGGGAG TTGG-3

Sc-R primer is 5-AGCAGTGTGGGGTTAGAT-3
