# Supplementary material for: SINE Retrotransposon variation drives Ecotypic disparity in natural populations of Coilia nasus
Source: Mob DNA. 2020 Jan 8;11:4. doi: 10.1186/s13100-019-0198-8 (PMC6951006; doi:10.1186/s13100-019-0198-8)
Supplement: Supplementary file 2 — Additional file 2: Table S2. The consensus sequence of SINE family from genome of C. nasus. [file 13100_2019_198_MOESM2_ESM.pdf]

**Additional Table 2 The consensus sequence of SINE family from genome of *C. nasus***

| Sequence (5’–3’) |
| --- |
| GGGCAGCTGTGGCCTAGTGGTTAGGGAGTTGGTCTTGCAATCGGAAGGTT<br>GCCGGTTCGAATCCCGCCCTACCCATGGTTGAGATGCCCTTGAGCAAGGC<br>ATCTAACCCCACACTGTTCCAGGGACTGTAACTGAAACCCTGTAAATATCT<br>GTAAGTCGCTCTGGATAAGAGCGTCAGCTAAGTGTAATGTAATGTAATGT<br>AATGTAA |
