# Supplementary material for: SINE Retrotransposon variation drives Ecotypic disparity in natural populations of Coilia nasus
Source: Mob DNA. 2020 Jan 8;11:4. doi: 10.1186/s13100-019-0198-8 (PMC6951006; doi:10.1186/s13100-019-0198-8)
Supplement: Supplementary file 3 — Additional file 3: Table S3. Primers specific to the flanking sequences of the SINE insertion loci used to detect insertion polymorphisms in C. nasus populations. [file 13100_2019_198_MOESM3_ESM.pdf]

Supple Table 3 Primers are specific to the flanking sequence of the SINE insertion locus, and used to detect the inserted polymorphism of populations in *C. nasus*.

Locus	Primer Direction	Sequence (5'–3')
Ls5	F	AGTATGCTTGTTCACGTTCA
	R	TTCGCTCTATTGCTCTGTGT
Ls29	F	ATTGAGCTGGTTCCTGTCATAACG
	R	GGTCATGTTGCCTGTGTGTCTTT
Ls40	F	CCATCGGAGAGCACGCAACTT
	R	ACCTGCCGCCTTCCAACTGA
Ls58	F	CCACCAGGTCTGTCAGTGTTGTT
	R	GTGCCAGGATGGAGGATGTCATT
Ls60	F	CCACCAGGTCTGTCAGTGTTGT
	R	TGTTGTGAATGTGTCAGCAGTAAGG
