# Supplementary material for: SINE Retrotransposon variation drives Ecotypic disparity in natural populations of Coilia nasus
Source: Mob DNA. 2020 Jan 8;11:4. doi: 10.1186/s13100-019-0198-8 (PMC6951006; doi:10.1186/s13100-019-0198-8)
Supplement: Supplementary file 4 — Additional file 4: Table S4. Types, origins and percent similarities of the tRNA-related portions of SINEs in C. nasus. [file 13100_2019_198_MOESM4_ESM.pdf]

Supple Table 4 Type, origin and percent of the tRNA-related portion of SINE in *C. nasus*.

tRNA type	Original gene	Percent (%)
Ala	tRNA-Ala gene	76
Val	tRNA-Val gene	3
Thr	tRNA-Thr gene	6
Pro	tRNA-Pro gene	6
Arg	tRNA-Arg gene	6
Ser	tRNA-Ser gene	3
