# Supplementary material for: SINE Retrotransposon variation drives Ecotypic disparity in natural populations of Coilia nasus
Source: Mob DNA. 2020 Jan 8;11:4. doi: 10.1186/s13100-019-0198-8 (PMC6951006; doi:10.1186/s13100-019-0198-8)
Supplement: Supplementary file 5 — Additional file 5 Table S5. Genes annotated in SINE-hit contigs from the migratory type. [file 13100_2019_198_MOESM5_ESM.pdf]

Gene Protein-or-Domain Score E-Value COG-ID Function-Description Code Functional-Categories;
CL7924.Contig1_27A BH2504_1 49.7 1e-06 COG0515 Serine/threonine protein kinase RTKL General function prediction only ; Signal transduction mechanisms ; Transcription ; Replication, recombination and repair ;
Unigene69860_27A NMA0945 53.5 9e-08 COG0382 4-hydroxybenzoate polyprenyltransferase and related prenyltransferases H Coenzyme transport and metabolism ;
Unigene14408_27A PA3295 47.4 7e-06 COG0537 Diadenosine tetraphosphate (Ap4A) hydrolase and other HIT family hydrolases FGR Nucleotide transport and metabolism ; Carbohydrate transport and metabolism ; General function prediction only ;
Unigene55357_27A YDR208w 50.4 4e-06 COG5253 Phosphatidylinositol-4-phosphate 5-kinase T Signal transduction mechanisms ;
Unigene1502_27A ECU09g0350 53.1 6e-07 COG5117 Protein involved in the nuclear export of pre-ribosomes JU Translation, ribosomal structure and biogenesis ; Intracellular trafficking, secretion, and vesicular transport ;
CL5755.Contig1_27A MT0180 48.9 8e-06 COG1463 ABC-type transport system involved in resistance to organic solvents, periplasmic component Q Secondary metabolites biosynthesis, transport and catabolism ;
CL4398.Contig1_27A SP2146 58.5 4e-08 COG3669 Alpha-L-fucosidase G Carbohydrate transport and metabolism ;
Unigene1733_27A mlr0349 100 1e-21 COG0513 Superfamily II DNA and RNA helicases LKJ Replication, recombination and repair ; Transcription ; Translation, ribosomal structure and biogenesis ;
Unigene2215_27A MT2905 52.0 9e-06 COG0532 Translation initiation factor 2 (IF-2; GTPase) J Translation, ribosomal structure and biogenesis ;
CL7631.Contig1_27A PH1798 50.4 8e-06 COG1196 Chromosome segregation ATPases D Cell cycle control, cell division, chromosome partitioning ;
Unigene10183_27A DR2600 48.1 4e-06 COG2340 Uncharacterized protein with SCP/PR1 domains S Function unknown ;
Unigene74808_27A ECU11g0470 63.2 1e-10 COG5059 Kinesin-like protein Z Cytoskeleton ;
Unigene56329_27A RSc0362_1 52.8 5e-07 COG1622 Heme/copper-type cytochrome/quinol oxidases, subunit 2 C Energy production and conversion ;
Unigene33505_27A RSp0884 48.5 9e-06 COG1674 DNA segregation ATPase FtsK/SpoIIIE and related proteins D Cell cycle control, cell division, chromosome partitioning ;
CL2821.Contig1_27A YAL005c 50.4 6e-06 COG0443 Molecular chaperone O Posttranslational modification, protein turnover, chaperones ;
Unigene34736_27A MA2500 49.3 8e-06 COG0607 Rhodanese-related sulfurtransferase P Inorganic ion transport and metabolism ;
Unigene17588_27A MT0180 48.9 4e-06 COG1463 ABC-type transport system involved in resistance to organic solvents, periplasmic component Q Secondary metabolites biosynthesis, transport and catabolism ;
CL2278.Contig2_27A alr7261 50.4 8e-07 COG1864 DNA/RNA endonuclease G, NUC1 F Nucleotide transport and metabolism ;
CL2278.Contig1_27A alr7261 50.4 2e-06 COG1864 DNA/RNA endonuclease G, NUC1 F Nucleotide transport and metabolism ;
Unigene25460_27A SPBC17A3.11 50.1 1e-06 COG5273 Uncharacterized protein containing DHHC-type Zn finger R General function prediction only ;
L2217.Contig1_27A L159324_1 50.1 2e-06 COG1705 Muramidase (flagellum-specific) NU Cell motility ; Intracellular trafficking, secretion, and vesicular transport ;
CL2054.Contig2_27A SPAPYUK71.03c 49.7 8e-06 COG5038 Ca2+-dependent lipid-binding protein, contains C2 domain R General function prediction only ;
CL314.Contig1_27A MT0018 59.7 2e-08 COG0515 Serine/threonine protein kinase RTKL General function prediction only ; Signal transduction mechanisms ; Transcription ; Replication, recombination and repair ;
Unigene74725_27A YDL140c 55.8 2e-08 COG0086 DNA-directed RNA polymerase, beta' subunit/160 kD subunit K Transcription ;
CL2364.Contig1_27A mll3243 47.8 5e-06 COG1100 GTPase SAR1 and related small G proteins R General function prediction only ;
Unigene75172_27A alr3912 50.8 6e-07 COG2988 Succinylglutamate desuccinylase E Amino acid transport and metabolism ;
Unigene1291_27A YLR419w 53.9 4e-07 COG1643 HrpA-like helicases L Replication, recombination and repair ;
Unigene71903_27A YBL056w_1 48.5 3e-06 COG0631 Serine/threonine protein phosphatase T Signal transduction mechanisms ;
Unigene52482_27A VNG0514C 64.3 8e-10 COG0419 ATPase involved in DNA repair L Replication, recombination and repair ;
Unigene41286_27A Cgl1939 51.2 4e-07 COG0532 Translation initiation factor 2 (IF-2; GTPase) J Translation, ribosomal structure and biogenesis ;
Unigene25508_27A SPBC13G1.10c 174 8e-44 COG1204 Superfamily II helicase R General function prediction only ;

Unigene15117_27A YPL174c 49.3 3e-06 COG5244 Dynactin complex subunit involved in mitotic spindle partitioning in anaphase B D Cell cycle control, cell division, chromosome partitioning ;
CL546.Contig1_27A YPO0469 104 4e-22 COG0484 DnaJ-class molecular chaperone with C-terminal Zn finger domain O Posttranslational modification, protein turnover, chaperones ;
Unigene55358_27A ECU03g0330 60.1 5e-09 COG5253 Phosphatidylinositol-4-phosphate 5-kinase T Signal transduction mechanisms ;
Unigene53389_27A SPAC17H9.09c 60.1 4e-09 COG1100 GTPase SAR1 and related small G proteins R General function prediction only ;
CL3537.Contig1_27A YBR264c 48.1 9e-06 COG1100 GTPase SAR1 and related small G proteins R General function prediction only ;
CL1753.Contig1_27A RSc1191 50.4 8e-06 COG2812 DNA polymerase III, gamma/tau subunits L Replication, recombination and repair ;
CL539.Contig4_27A ML2591 54.7 1e-06 COG1463 ABC-type transport system involved in resistance to organic solvents, periplasmic component Q Secondary metabolites biosynthesis, transport and catabolism ;
CL85.Contig2_27A DRA0210 53.1 7e-07 COG0747 ABC-type dipeptide transport system, periplasmic component E Amino acid transport and metabolism ;
Unigene28490_27A ML0826 50.1 1e-06 COG0423 Glycyl-tRNA synthetase (class II) J Translation, ribosomal structure and biogenesis ;
Unigene11330_27A DRA0212 47.8 9e-06 COG2377 Predicted molecular chaperone distantly related to HSP70-fold metalloproteases O Posttranslational modification, protein turnover, chaperones ;
Unigene9755_27A CC1315 73.6 5e-13 COG0346 Lactoylglutathione lyase and related lyases E Amino acid transport and metabolism ;
