# Supplementary material for: SINE Retrotransposon variation drives Ecotypic disparity in natural populations of Coilia nasus
Source: Mob DNA. 2020 Jan 8;11:4. doi: 10.1186/s13100-019-0198-8 (PMC6951006; doi:10.1186/s13100-019-0198-8)
Supplement: Supplementary file 6 — Additional file 6 Table S6. Genes annotated in SINE-hit contigs from the resident type. [file 13100_2019_198_MOESM6_ESM.pdf]

Gene Protein-or-Domain Score E-Value COG-ID Function-Description Code
Functional-Categories;
CL16430.Contig2_tangwq ECU11g0660 87.4 6e-18 COG0639 Diadenosine tetraphosphatase and
related serine/threonine protein phosphatases T Signal transduction mechanisms ;
CL16430.Contig1_tangwq SPBC16H5.07c 87.0 7e-18 COG0639 Diadenosine tetraphosphatase
and related serine/threonine protein phosphatases T Signal transduction mechanisms ;
Unigene3118_tangwq ECU04g0700 87.0 7e-18 COG0639 Diadenosine tetraphosphatase and
related serine/threonine protein phosphatases T Signal transduction mechanisms ;
Unigene3112_tangwq SPAC823.15 85.9 2e-17 COG0639 Diadenosine tetraphosphatase and
related serine/threonine protein phosphatases T Signal transduction mechanisms ;
Unigene42182_tangwq SPAC22H10.04 81.6 3e-16 COG0639 Diadenosine tetraphosphatase and
related serine/threonine protein phosphatases T Signal transduction mechanisms ;
Unigene4496_tangwq YDL134c 80.9 5e-16 COG0639 Diadenosine tetraphosphatase and related
serine/threonine protein phosphatases T Signal transduction mechanisms ;
Unigene41350_tangwq YDL188c 80.5 7e-16 COG0639 Diadenosine tetraphosphatase and related
serine/threonine protein phosphatases T Signal transduction mechanisms ;
CL1677.Contig2_tangwq MT3990 57.0 4e-07 COG0455 ATPases involved in chromosome
partitioning D Cell cycle control, cell division, chromosome partitioning ;
CL1677.Contig1_tangwq Rv2839c 56.6 5e-07 COG0532 Translation initiation factor 2
(IF-2; GTPase) J Translation, ribosomal structure and biogenesis ;
CL1484.Contig1_tangwq MT2905 56.6 5e-07 COG0532 Translation initiation factor 2
(IF-2; GTPase) J Translation, ribosomal structure and biogenesis ;
Unigene55710_tangwq Rv0171 55.8 8e-07 COG1463 ABC-type transport system involved in
resistance to organic solvents, periplasmic component Q Secondary metabolites
biosynthesis, transport and catabolism ;
Unigene62483_tangwq MT0180 55.8 8e-07 COG1463 ABC-type transport system involved in
resistance to organic solvents, periplasmic component Q Secondary metabolites
biosynthesis, transport and catabolism ;
Unigene4416_tangwq Cgl1939 54.3 2e-06 COG0532 Translation initiation factor 2 (IF-2;
GTPase) J Translation, ribosomal structure and biogenesis ;
CL9563.Contig1_tangwq ECU05g0070_2 216 2e-55 COG1752 Predicted esterase of the
alpha-beta hydrolase superfamily R General function prediction only ;
Unigene63419_tangwq YML059c_2 199 4e-50 COG1752 Predicted esterase of the alpha-beta
hydrolase superfamily R General function prediction only ;
CL1484.Contig2_tangwq SPCC4B3.04c_1 187 2e-46 COG0664 cAMP-binding proteins -
catabolite gene activator and regulatory subunit of cAMP-dependent protein kinases T
Signal transduction mechanisms ;
CL1484.Contig3_tangwq SPCC4B3.04c_2 185 8e-46 COG1752 Predicted esterase of the
alpha-beta hydrolase superfamily R General function prediction only ;
Unigene78492_tangwq lin0592 67.8 5e-12 COG0653 Preprotein translocase subunit SecA
(ATPase, RNA helicase) U Intracellular trafficking, secretion, and vesicular transport ;
Unigene67266_tangwq Cj0942c 65.1 3e-11 COG0653 Preprotein translocase subunit SecA
(ATPase, RNA helicase) U Intracellular trafficking, secretion, and vesicular transport ;
Unigene67265_tangwq jhp0723 64.3 5e-11 COG0653 Preprotein translocase subunit SecA
(ATPase, RNA helicase) U Intracellular trafficking, secretion, and vesicular transport ;
Unigene67264_tangwq CT701 63.5 9e-11 COG0653 Preprotein translocase subunit SecA
(ATPase, RNA helicase) U Intracellular trafficking, secretion, and vesicular transport ;
Unigene67263_tangwq HP0786 63.2 1e-10 COG0653 Preprotein translocase subunit SecA
(ATPase, RNA helicase) U Intracellular trafficking, secretion, and vesicular transport ;
Unigene67262_tangwq SPy1805 60.8 6e-10 COG0653 Preprotein translocase subunit SecA
(ATPase, RNA helicase) U Intracellular trafficking, secretion, and vesicular transport ;
Unigene42419_tangwq SP1702 60.8 6e-10 COG0653 Preprotein translocase subunit SecA
(ATPase, RNA helicase) U Intracellular trafficking, secretion, and vesicular transport ;
Unigene73614_tangwq CPn0841 60.1 1e-09 COG0653 Preprotein translocase subunit SecA
(ATPase, RNA helicase) U Intracellular trafficking, secretion, and vesicular transport ;
Unigene5532_tangwq Rv3876 47.4 8e-06 COG0455 ATPases involved in chromosome
partitioning D Cell cycle control, cell division, chromosome partitioning ;
CL9563.Contig5_tangwq MT3990 47.4 8e-06 COG0455 ATPases involved in chromosome
partitioning D Cell cycle control, cell division, chromosome partitioning ;
CL9563.Contig4_tangwq NMB0059 76.3 2e-14 COG0484 DnaJ-class molecular chaperone with
C-terminal Zn finger domain O Posttranslational modification, protein turnover, chaperones ;
Unigene71813_tangwq NMA0209 76.3 2e-14 COG0484 DnaJ-class molecular chaperone with
C-terminal Zn finger domain O Posttranslational modification, protein turnover, chaperones ;
Unigene70273_tangwq CT341 75.1 4e-14 COG0484 DnaJ-class molecular chaperone with
C-terminal Zn finger domain O Posttranslational modification, protein turnover, chaperones ;
Unigene68912_tangwq CPn0032 74.7 5e-14 COG0484 DnaJ-class molecular chaperone with
C-terminal Zn finger domain O Posttranslational modification, protein turnover, chaperones ;
Unigene66832_tangwq all0438_1 47.0 8e-06 COG0515 Serine/threonine protein kinase
RTKL General function prediction only ; Signal transduction mechanisms ; Transcription ;
Replication, recombination and repair ;
Unigene21098_tangwq L138452_1 47.0 8e-06 COG0515 Serine/threonine protein kinase

RTKL   General function prediction only ; Signal transduction mechanisms ; Transcription ; Replication, recombination and repair ;
Unigene63953_tangwq CAC1728_1   47.0   8e-06   COG0515 Serine/threonine protein kinase RTKL   General function prediction only ; Signal transduction mechanisms ; Transcription ; Replication, recombination and repair ;
